# Public Confidence in COVID-19 Prevention and Response in Bangladesh

**DOI:** 10.3389/fpubh.2021.783726

**Published:** 2022-02-03

**Authors:** Edris Alam, Kazi Abdur Rahman, Al-Ekram Elahee Hridoy

**Affiliations:** ^1^Faculty of Resilience, Rabdan Academy, Abu Dhabi, United Arab Emirates; ^2^Department of Geography and Environmental Studies, University of Chittagong, Chittagong, Bangladesh; ^3^The Government of the People's Republic Bangladesh, Dhaka, Bangladesh

**Keywords:** COVID-19, confidence, preparedness, response, risk management, Bangladesh

## Abstract

Community confidence in institutional approaches to emergency management directs how they cooperate and comply with public policy responses. In the context of emerging COVID-19 pandemic risk management, this study aims to assess public confidence in the Government of Bangladesh (GoB) and private sector entities for the activities undertaken during preparedness, prevention, and response phases. A survey was conducted with 307 respondents who willingly took part in the study. Cronbach's alpha was calculated to assess the internal reliability and the Mann-Whitney U test was conducted to estimate the mean score difference between the observations. A confirmatory factor analysis (CFA) was applied in the study. The findings suggest that the participants were highly positive about the GoB efforts to organize and provide PPE for doctors in time as a safeguard against COVID-19 and coordination and informed decision making in relation to facing COVID-19. Overall, the participants showed a lower-level confidence in the preparedness and response measures taken by authorities in Bangladesh. The results explored how the GoB failed to reach the public satisfaction level regarding provision of food and financial support to low income and middle income people. A lack of collaboration and coordination among different inter-GoB and private sectors makes mitigation and recovery process difficult. This research provides a set of policy recommendations for future public health emergency management based on the participants' concerns and suggestions, and a review of consequences of policy responses in the early stage.

## Introduction

The very first confirmed case of COVID-19 was registered in Bangladesh on March 8, 2020. According to the Government of Bangladesh (GoB), as of 23^rd^ May 2021, the number of confirmed infected cases and reported deaths in Bangladesh are 789,080 and 12,356, respectively (1). In response to the COVID-19 pandemic, the GoB has undertaken some non-medical and medical policy interventions such as ensuring limited gatherings for praying at mosques, social isolation, self-isolation, social distancing, quarantine, the opportunity to work from home, lockdown, travel restrictions, closure of non-essential services, flight restrictions, continuous disinfection, and sterilization initiatives. The GoB announced a nationwide holiday for 10 days from March 26, 2020 to April 4, 2020 which is recognized as “lockdown.” People were asked to avoid social gatherings and travel and to maintain good hygiene. The government of Bangladesh (GoB) deployed the army to ensure social distancing at that time. Based on the situation, the lockdown was prolonged up to May 30, 2020. The GoB has taken two different types of lockdown, namely partial and full lockdown. During the time of full lockdown, people were prohibited from going outside, except for emergency medical reasons. During partial lockdown, people were allowed to do their daily necessary activities and work duties while maintaining social distancing. Although several strategies and policies have been undertaken and implemented throughout Bangladesh with the aim of averting the negative effects of COVID-19, there exists a concern about the effectiveness of these measures. Several factors are responsible for this ineffectiveness such as late incorrect decision making, lack of knowledge about virus dissemination, lack of timely decisions and implementation, fake news and misinformation, and lack of resources (2, 3). Due to the lack of coordination and cooperation among various government institutions, non-government organizations, and the community, the GoB was unable to succeed completely in implementing lockdown for fighting against the pandemic (4).

It is difficult to prepare strategically or respond promptly when combating emerging or existing challenges when limited resources are available (4, 5). Public and private health services were completely inexperienced at the onset of the COVID-19 outbreak in Bangladesh. At that time, the country lacks substantial sectoral medical policy, strategy, resources, and regulatory frameworks which are essential to fight against the COVID-19 pandemic (2). Thermal scanners were installed in three international airports, namely Hazrat Shahjalal International Airport, Dhaka; Osmani International Airport, Sylhet; and in Shah Amanat International Airport, Chittagong, to measure the body temperature of a significant number of inbound passengers using infrared technology. Due to an inadequate number of testing kits, it was impossible to conduct a rapid test identifying an accurate number of confirmed COVID-19 cases. Healthcare staff and other frontline workers including the police, the army, and cleaners have not been supplied with adequate personal projecting equipment (PPE) to protect themselves from coronavirus infections (6). The country has only 5.3 doctors for every 10,000 residents, 0.3 nurses for every 1,000 people, 0.87 hospital beds for every 1,000 people, and 1.1 ventilators for every 100,000 population showing the fragility of the medical sector to the emerging threat of COVID-19 (7).

In this vulnerable situation, public engagement in prevention initiatives relies heavily on faith and confidence in the healthcare system. Trust is measured based on prior familiarity with the healthcare system. Confidence and belief in the health sector leads people to support and comply with public health programs (8). An individual's relationship with healthcare services relies strongly on faith (9). High level of awareness and knowledge are correlated with more favorable attitudes toward COVID-19 prevention activities (10–12). Bangladeshi people are highly superstitious and engulfed with false ideas about the virus spread and diagnostic procedures. It is time that the authorities start awareness campaigns to foster public confidence and ensure public participation and compliance with medical and non-medical policy measures (13). To being this campaign, the authority must know the existing confidence level of the people of Bangladesh in the GoB and private sectors for preparing for and responding to COVID-19. Limited research was conducted to assess the residents' confidence in preparedness and response measures undertaken by the GoB and private sectors at the early stage of the COVID-19 pandemic in Bangladesh. Thus, this study aims to examine Bangladeshi residents' confidence in preparedness and response measures undertaken by the GoB and private sectors between January and May 2020.

## Methods and Approaches

### Study Area and Data Collection Procedure

Bangladesh is a South Asian country inhabited by **166,423,708** people. The country has an area of 148,560 km^2^ positioned at 20° 30′ to 26° 45′ N latitude and 88° 0′ to 92° 45′ E longitude. Data was collected through questionnaires in electronic format dated between 12 April and 11 June 2020. A questionnaire based on structured questions was constructed and posted in the online Google Docs platform that was disseminated to the participants via e-mail and social media. Thus, a convenience sampling was applied to collect data from potential participants aged above 18. A total of 307 respondents participated in this study where significant respondents were from Dhaka and Chattogram. Participants were made aware of the purpose and context of this survey so that informed consent was confirmed. Anonymity of the respondents is ensured in this research. The questions used a 5-point Likert scale, ranging from 1 to 5 (strongly disagree, disagree, neutral, agree, and strongly agree).

### Questionnaire and Internal Reliability

A structured questionnaire was prepared to collect data on preparedness and response measures taken by the GoB and private sectors. The questionnaire consists of three major sections. The first section includes participant's socio demographic characteristics including age, gender, marital status, area of residence, sectors of work, income, and education levels. The second section containing 23 items focusing on participants' trust in preparedness and response measures taken by the GoB. The last part covers 5 items related to the preparedness, response and business continuity initiatives adopted by authorities of the private sector in Bangladesh between January and May 2020. An open-ended section was available in both section 2 and 3 for the participants to indicate whether they were unsatisfied with or mistrusted the activities of GoB and private sectors.

Internal reliability is a widely used metric that examines whether different items on the same test produce similar result based on correlations between them (14). Cronbach's alpha measure to test internal reliability has been utilized in this study. The Cronbach's alpha (∞) ranges between 0 and 1. This tool has been used by several researchers to identify inconsequential items in a questionnaire survey e.g., Emerson (15), Liu (16), Muhaimin et al. (17). However, the alpha score higher than 0.7 is desirable and an alpha score higher than 0.55 is acceptable (18).

Table 1 shows good values of Cronbach's alpha in government preparedness, and acceptable values for response effort and private sector's preparedness. In summary, the internal consistency of the data is satisfactory.

**Table 1:** Items in each factor and their internal reliability.

**Variable**	**Items in each factors**	**Cronbach's alpha**
**Factor 1: Government preparedness**		
GP1	The government of Bangladesh held the information about the immigrants and database of the immigrants and organized quarantine arrangements for the foreign visitors in time as a safeguard against COVID-19.	0.789
GP2	The government of Bangladesh organized home quarantine/separate arrangements/isolation for repatriates Bangladeshi in time as a safeguard against COVID-19.	0.779
GP3	The government of Bangladesh organizes sterilization of non-passenger's goods/product/items that arrived from overseas in time as a safeguard against COVID-19.	0.787
GP4	The government of Bangladesh organized countrywide sterilization and disinfection activities in time as a safeguard against COVID-19.	0.779
GP5	The government of Bangladesh organizes testing support for the suspects of COVID-19 infected patient in time as a safeguard against COVID-19.	0.771
GP6	The government of Bangladesh organized and prepared hospitals for affected people in time as a safeguard against COVID-19.	0.775
GP7	The government of Bangladesh organized and provided PPE for doctors in time as a safeguard against COVID-19.	0.798
GP8	The government of Bangladesh organized food and support for the low-income people in time.	0.798
GP9	The government of Bangladesh organized food and support for the middle-income people and other people who become jobless during COVID-19 pandemic.	0.858
GP10	There are lacking in coordination and decision making exists in relation to facing COVID-19?	0.792
GP11	The government entities in Bangladesh have organized basic health services, food management and education facilities (through distance/online learning) to deal with the consequences of the epidemic diseases such as COVID-19 pandemic.	0.786
**Factor 2: Response effort**		
RE1	All the agencies within the government of Bangladesh have prepared response and business continuity plans to deal with the consequences of epidemic diseases such as COVID-19 pandemic in time.	0.625
RE2	All the preventive, responsive, and business continuity plans prepared by the different agencies within the government of Bangladesh will be able to deal with the consequences of epidemic diseases such as COVID-19 pandemic in time.	0.591
RE3	All the government agencies within the government of Bangladesh have the required capabilities to deal with the consequences of COVID-19 pandemic and ensure the business continuity through providing services by implementing the business continuity plans.	0.621
RE4	The agencies within the government of Bangladesh are successful in implementing the media response plans for risk communication providing accurate and timely information about COVID-19.	0.613
RE5	A sample was taken from the community to develop the response and business continuity plans in the various government sectors to insure the suitability of these plans during epidemic diseases such as COVID-19 pandemic.	0.623
RE6	The government entities in Bangladesh have the flexibility to deal with the consequences of the epidemic diseases such as COVID-19 pandemic.	*0.644*
RE7	The Government of Bangladesh is one of the model countries in dealing with COVID-19 pandemic and business continuity?	0.660
RE8	Do you think that some of the procedures and services (i.e., work from home, online meeting, tele-prescription, online services provision etc.) provided by the government entities in Bangladesh to cope with COVID-19 pandemic will be continuing in the future and change government service delivery mechanism?	0.677
**Factor 3: Private sector's preparedness**		
PP1	All the private organizations in Bangladesh have prepared response and business continuity plans to deal with the consequences of epidemic diseases such as COVID-19 pandemic in time.	0.601
PP2	All the preventive, responsive, and business continuity plans prepared by the different private organizations in Bangladesh will be able to deal with the consequences of epidemic diseases such as COVID-19 pandemic.	0.682
PP3	A sample was taken from the community to develop the response and business continuity plans in the private organizations from various sectors to insure the suitability of these plans during epidemic diseases such as COVID-19 pandemic.	0.652
PP4	The private entities within Bangladesh have the flexibility to deal with the consequences of the epidemic diseases such as COVID-19 pandemic.	0.663
PP5	Do you think that some of the procedures and services (i.e., work from home, online meeting, tele-prescription, and online services provision etc.) provided by the private entities in Bangladesh to cope with COVID-19 pandemic will be continuing in the future and change private sector service delivery mechanism?	0.627

### Statistical Analysis

Likert data was compared using Mann–Whitney-U test for two groups: (i) Male and female and (ii) Government and Private sectors to compare if the response variable deviates between these two groups (19–21). Further, factor analysis determines the underlying factors or latent variables among the questionnaire items or observed variables. Factor analysis can be two types: exploratory factor analysis (EFA) and confirmatory factor analysis (CFA). For this study, EFA was eliminated as it is more data-driven regarding hypothesized measurement model explicitly to be examined (22, 23). Moreover, this method also has been used to perceive the people's perception in different aspect during COVID-19 situation (24, 25). Particular variables relate to factors; thus, CFA is employed in this study (26). However, an ideal standardized factor loading should be higher than 0.5, and ideally 0.7 or higher. Therefore, a revised CFA model was derived by eliminating several variables with standardized factor loadings lower than 0.5.


(1) $$\begin{array}{l} Y=\wedge \xi +\epsilon \end{array}$$

The observed parameters are Y, the unobserved latent constructs are ξ, the number of factor loadings is ∧, and the probabilistic case is estimated by ϵ iteratively lessening the fit function (27).

The sample size needed for CFA should be at least 200–300 participants (28). The CFA was performed on the three factors: government preparedness and response effort, and private sector preparedness. The CFA is utilized to test the fit between the measurement model and actual data. Here, the measurement model was assessed by the following Tucker-Lewis's index (TLI), Comparative Fit Index (CFI), and root-mean-square error of approximation (RMSEA) to fit the conventional cutoff principles (29–31). TLI, CFI, and RMSEA are defined based on the fit function. The statistical equations for the above three tools are as follows, following (32):


(2) $$\begin{array}{l} TLI=1-\frac{\frac{F_{H}}{df_{H}}}{\frac{F_{B}}{df_{B}}} \end{array}$$

(3) $$\begin{array}{l} CFI=1-\frac{F_{H}}{F_{B}} \end{array}$$

(4) $$\begin{array}{l} RMSEA=\sqrt{\frac{F_{H}}{df_{H}}} \end{array}$$

The significance level (*p* = < 0.001) is considered for factor loadings. Where, base model and hypothesized model are, respectively, indicated by H and B. Both *F*_*H*_and *F*_*B*_ are the minimized fit function of the respective models. Model degrees of freedom denoted by *df*_*H*_ and *df*_*B*_.

## Results

### Socio-Demographic Characteristics of the Participants

The socio-demographic characteristics of the participants have been shown in Table 2. Out of 307 participants, 236 were male and 71 were female. There exists age variation among the participants. Three age groups are identified. In total, 64.8, 33.2, and 2% of participants are in the age groups of 18–30, 31–50, and 51–65 years, respectively. Most of the participants have completed a master's degree (45.6%), followed by a bachelor's degree (41%), doctor of philosophy (PhD) (6.2%), higher secondary school certificate (HSC) (5.9%), diploma (1%), and secondary school certificate (SSC) (0.3%). The percentage of participants working for public and private sectors were 63.5 and 36.5%, respectively (Table 2).

**Table 2:** Socio-demographic characteristics of the participants.

**Items/Characteristics of the participants**	**Frequency (%)**
**Gender**	
Male	236 (76.9)
Female	71 (23.1)
**Marital status**	
Single	174 (56.7)
Married	130 (42.3)
Preferred not to tell	3 (1)
**Age group**	
18–30	199 (64.8)
31–50	102 (33.2)
51–65	6 (2)
**Place of living**	
Dhaka	148 (48.2)
Chattogram	118 (38.4)
Khulna	14 (4.6)
Rajshahi	11 (3.6)
Mymensnigh	9 (2.9)
Barishal	4 (1.3)
Sylhet	3 (1.0)
**Education level**	
SSC	1 (0.3)
HSC	18 (5.9)
Diploma	3 (1.0)
Bachelor degree	126 (41)
Master degree	140 (45.6)
Doctor of Philosophy (PhD)	19 (6.2)
**Sector of work**	
Public	112 (36.5)
Private	195 (63.5)

### Descriptive Statistics of the Variables

Descriptive statistics of the variables including mean, standard deviation, variance, skewness, and kurtosis have been measured. A summary of the initiated variables is provided in Table 3. The highest mean, standard deviation, variance, skewness, and kurtosis values are 3.74 (RE7), 1.22 (GP5 and GP6), 1.58 (GP2), 1.38 (GP10), and 1.7 (GP10), respectively.

**Table 3:** Descriptive statistics of the variables.

**Variables**	**Mean**	**Std. Dev**	**Variance**	**Skewness**	**Kurtosis**
GP1	2.93	1.18	1.4	0.107	−1.22
GP2	2.87	1.26	1.58	0.119	−1.28
GP3	3.18	1.08	1.18	−0.277	0.655
GP4	3.29	1.22	1.49	−0.324	−1.02
GP5	3	1.22	1.5	0.211	−1.2
GP6	3.07	1.22	1.49	−0.0176	−1.26
GP7	3.46	1.14	1.3	−0.261	−1.04
GP8	2.52	1.02	1.04	0.312	−0.92
GP9	3.41	1.1	1.2	−0.543	−0.558
GP10	1.92	1.01	1.03	1.38	1.7
GP11	3.07	1.12	1.26	0.17	−1.01
RE1	3.36	1.11	1.22	−0.221	−1.06
RE2	3.13	0.997	0.993	−0.2	−0.958
RE3	3.41	0.982	0.965	−0.501	−0.474
RE4	3.13	1.06	1.12	0.211	−1.23
RE5	3	1.01	1.03	0.106	−0.757
RE6	3.15	1.06	1.12	−0.0313	−0.962
RE7	3.74	1.16	1.35	−1.03	0.373
RE8	2.56	0.975	0.951	0.86	0.0483
PP1	3.3	1.1	1.2	−0.672	−0.521
PP2	3.14	1.07	1.15	−0.424	−0.734
PP3	3.13	0.96	0.921	−0.442	−0.576
PP4	3.08	1.07	1.14	0.0643	−1.06
PP5	2.45	0.837	0.7	1.25	1.65

### Confirmatory Factor Analyses Model

The initial CFA model of three factors (government preparedness, response effort, and private sector preparedness) is shown in Table 4. The factor loadings with its *p*-value for each item have been included in the Table 4. An ideal standardized factor loading should be higher than 0.5 and ideally 0.7 or higher (33).

**Table 4:** Initial factor loadings and *P*-value of the items.

**Factors**	**Variable**	***p*** **-value**	**Stand. estimate**	**Included/excluded**
Government preparedness	GP1	<0.001	0.6238	Included
	GP2	<0.001	0.6392	Included
	GP3	<0.001	0.6851	Included
	GP4	<0.001	0.7548	Included
	GP5	<0.001	0.6604	Included
	GP6	<0.001	0.7343	Included
	GP7	<0.001	0.6866	Included
	GP8	<0.001	0.4171	Excluded
	GP9	<0.001	0.4123	Excluded
	GP10	<0.001	−0.2786	Excluded
	GP11	<0.001	0.5635	Included
Response effort	RE1	<0.001	0.5224	Included
	RE2	<0.001	0.6212	Included
	RE3	<0.001	0.5544	Included
	RE4	<0.001	0.4921	Excluded
	RE5	<0.001	0.5666	Included
	RE6	<0.001	0.4585	Excluded
	RE7	<0.001	0.4295	Excluded
	RE8	0.007	0.1703	Excluded
Private sector preparedness	PP1	<0.001	0.7845	Included
	PP2	<0.001	0.8276	Included
	PP3	<0.001	0.5873	Included
	PP4	0.133	0.0951	Excluded
	PP5	0.093	0.1069	Excluded

Therefore, a revised CFA model was derived by eliminating several variables which had standardized factor loadings lower than 0.5. The eliminated variables are GP8, GP9, GP10, RE4, RE6, RE7, RE8, PP4, and PP5. The final CFA of public confidence on risk management toward government and private sectors is presented in Table 5. Table 6 represents the parameter estimates and goodness of fit measures of the final model. It is apparent that CFI, TLI, and RMSEA values are 0.932, 0.920, and 0.070, respectively. It indicates the results found from the model CFA are acceptable.

**Table 5:** Revised factor loading and *P*-value of each variable.

**Factor**	**Variable**	***p*** **-value**	**Stand. estimate**
Government Preparedness	GP1	<0.001	0.620
	GP2	<0.001	0.650
	GP3	<0.001	0.682
	GP4	<0.001	0.770
	GP5	<0.001	0.671
	GP6	<0.001	0.736
	GP7	<0.001	0.678
	GP11	<0.001	0.539
Response Effort	RE1	<0.001	0.522
	RE2	<0.001	0.715
	RE3	<0.001	0.554
	RE5	<0.001	0.618
Private Sector Preparedness	PP1	<0.001	0.789
	PP2	<0.001	0.829
	PP3	<0.001	0.586

**Table 6:** Goodness of fit measures of CFA model.

**Fit indices**	**Parameter estimates**	
	**Initial**	**Final**
Comparative Fit Index (CFI)	0.685	0.932
Tucker Lewis Index (TLI)	0.651	0.920
Root Mean Square Error of approximation (RMSEA)	0.102	0.070

### Difference in Mean Items Scores Between Male and Female Groups

A Mann-Whitney U test has been performed to assess if the observation of any randomly drawn sample is larger than the other or the distributions are equal. A Mann-Whitney U test was conducted to measure the mean scores difference between the study variables of male and female groups and government and private sectors sample (Tables 7, 8). The test results suggest that there are statistically significant differences in the mean scores of GP2 and GP3, GP4, GP5, GP6, GP8, GP11, and RE8 on male and female groups, respectively. Equal observations are measured for the remaining variables (Table 7).

**Table 7:** Test results of Mann-Whitney U test on female and male groups.

**Variables**	**Group**	**N**	**Mean**	***p*** **-value**
GP1	Female	71	2.97	0.089
	Male	236	3.24	
GP2	Female	71	2.62	0.018
	Male	236	3.03	
GP3	Female	71	3.04	0.039
	Male	236	3.35	
GP4	Female	71	2.99	0.014
	Male	236	3.41	
GP5	Female	71	2.80	0.004
	Male	236	3.29	
GP6	Female	71	2.87	0.008
	Male	236	3.31	
GP7	Female	71	3.24	0.060
	Male	236	3.55	
GP8	Female	71	2.46	0.010
	Male	236	2.81	
GP9	Female	71	3.39	0.759
	Male	236	3.47	
GP10	Female	71	2.00	0.337
	Male	236	1.93	
GP11	Female	71	2.86	0.011
	Male	236	3.24	
RE1	Female	71	3.25	0.348
	Male	236	3.39	
RE2	Female	71	3.20	0.521
	Male	236	3.14	
RE3	Female	71	3.38	0.772
	Male	236	3.41	
RE4	Female	71	2.92	0.120
	Male	236	3.13	
RE5	Female	71	2.89	0.538
	Male	236	2.96	
RE6	Female	71	3.38	0.128
	Male	236	3.17	
RE7	Female	71	3.66	0.228
	Male	236	3.79	
RE8	Female	71	2.49	0.047
	Male	236	2.74	
PP1	Female	71	3.15	0.713
	Male	236	3.21	
PP2	Female	71	3.07	0.919
	Male	236	3.05	
PP3	Female	71	3.06	0.371
	Male	236	2.94	
PP4	Female	71	3.01	0.619
	Male	236	3.09	
PP5	Female	71	2.52	0.929
	Male	236	2.49	

**Table 8:** Test results of Mann-Whitney U test on government and private sector groups.

**Variables**	**Group**	***N***	**Mean**	***p*** **-value**
GP1	Government Sector	112	2.93	0.006
	Private Sector	195	3.32	
GP2	Government Sector	112	2.87	0.493
	Private Sector	195	2.97	
GP3	Government Sector	112	3.18	0.299
	Private Sector	195	3.34	
GP4	Government Sector	112	3.29	0.806
	Private Sector	195	3.33	
GP5	Government Sector	112	3.00	0.057
	Private Sector	195	3.28	
GP6	Government Sector	112	3.07	0.127
	Private Sector	195	3.29	
GP7	Government Sector	112	3.46	0.709
	Private Sector	195	3.49	
GP8	Government Sector	112	2.52	0.015
	Private Sector	195	2.85	
GP9	Government Sector	112	3.41	0.481
	Private Sector	195	3.47	
GP10	Government Sector	112	1.92	0.494
	Private Sector	195	1.96	
GP11	Government Sector	112	3.07	0.373
	Private Sector	195	3.20	
RE1	Government Sector	112	3.36	0.903
	Private Sector	195	3.36	
RE2	Government Sector	112	3.13	0.777
	Private Sector	195	3.16	
RE3	Government Sector	112	3.41	0.795
	Private Sector	195	3.39	
RE4	Government Sector	112	3.13	0.646
	Private Sector	195	3.06	
RE5	Government Sector	112	3.00	0.516
	Private Sector	195	2.91	
RE6	Government Sector	112	3.15	0.468
	Private Sector	195	3.26	
RE7	Government Sector	112	3.74	0.583
	Private Sector	195	3.77	
RE8	Government Sector	112	2.56	0.097
	Private Sector	195	2.75	
PP1	Government Sector	112	3.30	0.100
	Private Sector	195	3.13	
PP2	Government Sector	112	3.14	0.148
	Private Sector	195	3.01	
PP3	Government Sector	112	3.13	0.010
	Private Sector	195	2.88	
PP4	Government Sector	112	3.08	0.888
	Private Sector	195	3.07	
PP5	Government Sector	112	2.45	0.780
	Private Sector	195	2.53	

### Difference in Mean Items Scores Between Government and Private Sector Groups

A Mann-Whitney U test has also been conducted for the observation of government and private sectors to explore the difference in the mean score between those two distributions. It is evident that there were statistically significant differences in the mean scores of GP1, GP8, and PP3 variables (Table 8). The rest of the observations follow the null hypothesis that there is equal probability of exceeding one random observation than the other.

## Discussion

The research was based on the perception of Bangladeshi citizens of risk management against COVID-19. The CFA result suggests that GP4 “The government of Bangladesh organized countrywide sterilization and disinfection activities in time as a safeguard against COVID-19” was the most significant variable for Governmental preparedness. For response effort RE2 “All the preventive, responsive, and business continuity plans prepared by the different agencies within the government of Bangladesh will be able to deal with the consequences of epidemic diseases such as COVID-19 pandemic in time.” was found as the most significant variable for response effort. For private sector preparedness, “All the preventive, responsive, and business continuity plans prepared by the different private organizations in Bangladesh will be able to deal with the consequences of epidemic diseases such as COVID-19 pandemic” was determined as the most important variable.

From the Mann-Whitney U test, the findings suggest statistically significant variations in the mean scores of GP2, GP3, GP4, GP5, GP6, GP8, GP11, and RE8. Thus, there was a disparity between the perceptions of males and females. Moreover, there were significant variations in the mean score of the GP1, GP8, and PP3 metrics which indicate that there exists distinctive perception among participants working for the government and private sectors.

A high degree of public confidence in institutions and a low level of perceived danger is a favorable state in normal conditions. Public confidence established on the impression of integrity, caring and transparency of the government might cause citizens to overlook risks and therefore minimize their conviction in the need to undertake individual measures to manage risks (8). However, in the case of a pandemic like COVID-19, public enforcement is difficult. As part of the findings, the government of Bangladesh arranged sterilization of non-passenger goods/products/items that arrived from overseas, as well as food and support for low-income citizens as a precaution against COVID-19. This calls into question the risk research presumption that open communication, especially of complexities, enables the community to make critical choices (34).

An updated CFA model was estimated. Few variables such as GP8, GP9, GP10, RE4, RE6, RE7, RE8, PP4 and PP5 were eliminated because their uniform factor loadings were < 0.5. It is also noted that the GoB had coordinated national sterilization and disinfection activities as a precaution against COVID-19, which was important in the risk management of COVID-19. However, in terms of the organization of essential health services, food management, and education facilities (via distance/online learning), Bangladeshi government institutions could deal with the implications of the COVID-19 pandemic. The population was pessimistic that Bangladesh had the flexibility to cope with the effects of infectious diseases. Good governance can only be accomplished by an intelligent early warning system, accurate review of the situation, understanding, exchange, and use of functional expertise and intelligence (35). However, the most striking aspect was the lack of coordination and decision-making for the government's COVID-19 situation. It was an unexpected situation for Bangladesh, like many other nations. The government was not ready enough to deal with a crisis that it never experienced in the past. The findings are consistent with those of (36–38).

It has been noted that, instead of monitoring the rumors and misinformation, propaganda, and hate speech, the GoB cracks down on those who condemn its treatment of the ongoing social media crisis. It has had a detrimental effect on public trust (39). There was a positive alliance of confidence that prevention, sensitivity, and business continuity strategies planned by the various private organizations in Bangladesh could cope with the effects of infectious diseases such as COVID-19. The GoB claimed that private organizations played an important role in the distribution of preventive messages during COVID-19 and collaborated closely with local authorities and government officials in the planning/management of quarantine/isolation centers. However, the findings of this research are in contrast with the government's argument. The outcome has shown that private companies within Bangladesh do not have the flexibility to cope with the effects of infectious diseases such as COVID-19.

## Conclusions

The findings suggest that the initiatives taken for COVID-19 by governmental and private entities were not sufficient. The GoB has adopted several major practices to combat this crisis. In comparison, the private sectors do not have such flexibility or capacity to combat with the current pandemic situation. The GoB should either facilitate the operations of private sectors and NGOs, or consider the lack of complete flexibility of non-governmental sectors and act accordingly. Nevertheless, private sectors should step forward with their highest capacity and fight the pandemic situation along with the mainstream governmental initiatives. A monitoring body for activities in the private sector might be beneficial for enhancing the public trust in the private sector. Policy makers should specially consider these phenomena, regarding the functional, capacitive, and operational difference between the public and private sectors while making the policy. Pandemic influenza risk management policy should not be constant since the situation is fluctuating frequently. Policies and initiatives should be adopted with recent studies and data. The only way to establish a strong public trust is to develop an effective and coordinative system between the public and private sectors in terms of healthcare, consistent social distancing policy implementation with sustained livelihood options for those in need, and other emergency service provisions for all.

## Data Availability Statement

The datasets presented in this study can be found in online repositories. The names of the repository/repositories and accession number(s) can be found in the article/supplementary material. The data presented in the study are deposited at: https://docs.google.com/spreadsheets/d/1odIZzwyTGgSj3kpPZ-YpoXCSiPV8KXjQgEVnL4Uqw1w/edit?fbclid=IwAR1mXpJkDH2SPBJarFU4s6yXKtq6DbB5C8EpOORUdGb-p8fB79uhznMgv_0#gid=0.

## Ethics Statement

The studies involving human participants were reviewed and approved by Institutional Review Board, University of Chittagong. The patients/participants provided their written informed consent to participate in this study.

## Author Contributions

EA, KR, and A-EH: conceptualization, validation, investigation, writing—original draft preparation, and writing—review and editing. EA and A-EH: formal analysis, methodology, data curation, and visualization. EA and KR: supervision and resources. All authors contributed to the article and approved the submitted version.

## Conflict of Interest

The authors declare that the research was conducted in the absence of any commercial or financial relationships that could be construed as a potential conflict of interest.

## Publisher's Note

All claims expressed in this article are solely those of the authors and do not necessarily represent those of their affiliated organizations, or those of the publisher, the editors and the reviewers. Any product that may be evaluated in this article, or claim that may be made by its manufacturer, is not guaranteed or endorsed by the publisher.
